# Intrinsic Human Elimination Half-Lives of Polychlorinated Biphenyls Derived from the Temporal Evolution of Cross-Sectional Biomonitoring Data from the United Kingdom

**DOI:** 10.1289/ehp.1002211

**Published:** 2010-10-08

**Authors:** Roland Ritter, Martin Scheringer, Matthew MacLeod, Claudia Moeckel, Kevin C. Jones, Konrad Hungerbühler

**Affiliations:** 1 Safety and Environmental Technology Group, ETH Zurich, Zurich, Switzerland; 2 Lancaster Environment Centre, Lancaster University, Lancaster, United Kingdom

**Keywords:** elimination half-life, exposure analysis, PCB, persistent organic pollutants, pharmacokinetic model

## Abstract

**Background:**

Most empirical estimates of human elimination kinetics for persistent chemicals reflect apparent elimination half-lives that represent the aggregated effect of intrinsic elimination, ongoing exposure, and changes in body weight. However, estimates of intrinsic elimination at background levels are required for risk assessments for the general population.

**Objective:**

To estimate intrinsic human elimination half-lives at background levels for nine polychlorinated biphenyl (PCB) congeners, we used a novel approach based on population data.

**Methods:**

We used a population pharmacokinetic model to interpret two sets of congener-specific cross-sectional age–concentration biomonitoring data of PCB concentrations measured in lipid and blood samples that were collected from 229 individuals in 1990 and 2003. Our method is novel because it exploits information about changes in concentration in the human population along two dimensions: age and calendar time.

**Results:**

Our approach extracted information about both elimination kinetics and exposure trends from biomonitoring data. The longest intrinsic human elimination half-lives estimated in this study are 15.5 years for PCB‐170, 14.4 years for PCB‐153, and 11.5 years for PCB‐180.

**Conclusions:**

Our results are further evidence that a maximum intrinsic elimination half-life for persistent chemicals such as PCBs exists and is approximately 10–15 years. A clear conceptual distinction between apparent and intrinsic half-lives is required to reduce the uncertainty in elimination half-lives of persistent chemicals. The method presented here estimates intrinsic elimination half-lives and the exposure trends of persistent pollutants using cross-sectional data available from a large and growing number of biomonitoring programs.

Persistent organic chemicals such as polychlorinated biphenyls (PCBs) and chlorinated dibenzodioxins and dibenzofurans may cause human health effects as a result of exposure that occurred years before effects manifest (Cohn et al. 2007). To reconstruct past exposures and relate exposure and body concentrations, pharmacokinetic (PK) models are increasingly used (Karmaus et al. 2004; Redding et al. 2008; Verner et al. 2008, 2009). The accuracy of reconstructed exposures or body concentrations of these chemicals depends on the availability and quality of estimates of the elimination half-life, which describes the removal of the chemical from the body by metabolic and nonmetabolic pathways.

Elimination kinetics in humans have been estimated from two major categories of biomonitoring data (Table 1): sequential measurements in the same individual, referred to as longitudinal data (LD); and population biomonitoring data from many individuals at one point in time, called cross-sectional data (CSD). Most elimination half-lives reported in the literature are estimated from concentration declines in LD for particular individuals. This method assumes that ongoing exposure during the declining phase is negligible (Phillips 1989). However, for persistent chemicals with long elimination half-lives, study participants cannot be isolated from ongoing exposure during the decline phase of an experiment, for example, by fasting (Koch and Angerer 2007). Shirai and Kissel (1996) recognized this confounding factor and introduced the term “apparent” elimination half-life, as opposed to “true” elimination half-life, to refer to experimentally observed elimination half-lives that may be affected by ongoing exposure. In addition, because of the long intervals (several years) required in LD-based studies to measure persistent chemicals, changes in body weight are an additional factor that influences concentrations over time (Grandjean et al. 2008; Yakushiji et al. 1984). Thus, the definition of apparent elimination half-life that was used by Shirai and Kissel (1996) has been extended to include the combined effect of elimination, ongoing exposure, and changes in body composition (Milbrath et al. 2009).

However, apparent half-lives cannot be used to parameterize elimination in a PK model because they describe the observed decline in concentration under specific conditions of ongoing exposure and changes in body size and composition. Apparent half-lives that have been reported for persistent chemicals such as PCBs and polychlorinated dibenzodioxins and dibenzofurans are therefore highly variable. Values for individual substances range from < 1 year to several decades, and even negative values have been reported (Matsumoto et al. 2009; Milbrath et al. 2009; Shirai and Kissel 1996). We use the term “intrinsic” half-life, rather than “true” half-life (Shirai and Kissel 1996), to unequivocally specify half-lives estimated with methods that account for, and thereby eliminate the influence of, the effects of ongoing exposure and changes in body weight. Correspondingly, and in accordance with previous authors, we use “apparent half-life” to refer to half-life estimates that directly reflect the observed change in concentration in one individual over time that is determined mainly by the aggregated effect of intrinsic elimination, ongoing exposure, and body weight changes, although additional factors such as smoking habits and parity may also have an influence.

Estimating intrinsic elimination half-lives of persistent chemicals therefore requires correcting for effects other than intrinsic elimination (i.e., changes in body weight and ongoing exposure). A marked increase in body weight occurs during childhood and causes “growth dilution” of chemical concentrations in the body. Growth dilution has been accounted for in LD-based studies of children (Grandjean et al. 2008; Yakushiji et al. 1984). Accounting for ongoing exposure in LD-based studies requires exposure estimates for every individual. In an LD-based study from the Faroe Islands, Grandjean et al. (2008) accounted for ongoing exposure by including the number of meals of whale meat consumed by each subject as a covariate. However, most LD-based studies do not account for ongoing exposure but instead use individuals from occupationally or accidentally exposed cohorts (“incident cohorts”) who experienced high exposures for a restricted time period so that background exposure could be considered negligible (Shirai and Kissel 1996). However, these estimates are not representative of the general population because intrinsic elimination reflects the individual status of the patient and has been shown to be faster and partly driven by different elimination mechanisms at high concentrations, such as induced metabolism or increased elimination via skin and feces (Sorg et al. 2009). Because of confounding from ongoing exposure, estimation of elimination kinetics from cohorts at background concentration has even been judged to be impossible (Lotti 2003; Yakushiji et al. 1984).

To avoid the difficulty of accounting for ongoing exposure in LD-based studies of cohorts at background levels, alternative approaches based on CSD have been proposed (Table 1, rows 3–7). The simplest approach is based on one average body concentration derived from a set of CSD and relates this concentration to one average estimate of dietary intake by using a PK model under the assumption of steady state (constant intake, constant elimination; Table 1, row 3) (Geyer et al. 2004; Ogura 2004; Shirai and Kissel 1996). We recently demonstrated a more sophisticated method (Ritter et al. 2009) that accounts for decreasing population exposure in a postban phase and uses multiple concentrations averaged over the population from several CSD collected at different times, which are referred to as cross-sectional trend data (CSTD; Table 1, row 4). Both of these CSD-based approaches use average CSD.

Cross-sectional studies usually also collect information about age and other variables to complement information about the body concentration of pollutants. A few studies have exploited the information in the cross-sectional age–concentration relationship to estimate intrinsic human elimination half-lives for PCBs, dioxins, and furans. These studies used one set of “age–concentration CSD” representing one specific year and detailed empirical knowledge about the historic exposure trend (Ogura 2004; Van der Molen et al. 2000; Table 1, row 5).

To our knowledge, no study exists that uses more than one set of empirical age– concentration CSD collected in different years to estimate intrinsic elimination half-lives. Such an approach uses information from concentration changes along two temporal dimensions in combination: the age–concentration relationship at a given time within each set of CSD (Ogura 2004; Van der Molen et al. 2000) and the cross-sectional trend as a function of calendar time (Ritter et al. 2009). Here we use two sets of age–concentration CSD and pursue three main goals: first, to provide estimates of intrinsic elimination half-lives from the human body at background exposure levels for nine PCB congeners; second, to compare half-life estimates with literature data to discuss plausible ranges for intrinsic elimination half-lives compared with apparent half-lives; and third, to evaluate the possibility to access information about historic exposure contained in the temporal evolution of the age–concentration relationship in cross-sectional population biomonitoring data.

## Materials and Methods

### Empirical data

We use two sets of congener-specific cross-sectional biomonitoring data for PCBs. The first set consists of PCB concentration in 75 adipose tissue samples from Wales, United Kingdom, which were collected in 1990–1991 (Duarte-Davidson et al. 1994). The age of the individuals who supplied the samples ranged from 14 to 79 years. The second set consists of 154 human blood samples collected in 2003 at 13 locations in the United Kingdom (Thomas et al. 2006). The age of these individuals ranged from 22 to 80 years. To reduce the influence of outliers, we aggregated the data in 10 age groups (in both data sets). Both studies reported results in terms of lipid-normalized concentrations. Lipid-normalized concentrations derived from blood and adipose tissue samples are directly comparable because the two lipid compartments are in equilibrium (Haddad et al. 2000; Patterson et al. 1988; Sorg et al. 2009). Details about samples and analytical methods have been described elsewhere for both data sets (Duarte-Davidson et al. 1994; Thomas et al. 2006).

Congener-specific empirical daily intake data for the U.K. population were derived from total diet studies because dietary intake is the main source of PCB exposure for the general population [for detailed description and references, see Supplemental Material (doi:10.1289/ehp.1002211)].

### Population PK model

We employed a population PK model that describes changes in body concentration of PCBs as a function of age and calendar time for multiple individuals representing different birth cohorts of the average population (Alcock et al. 2000; Pinsky and Lorber 1998). The model is a modified version of the multiindividual PK framework that was recently presented and analytically solved for a postban period (Ritter et al. 2009). The model implementation used here differs in two aspects from the earlier version: It is solved numerically and therefore is not restricted to postban conditions; and we implemented age-dependent growth of body mass and lipid mass, and age- and body-weight-dependent dietary intake, including intake by breast-feeding.

Equation 1 defines the time course of PCB concentration in one representative individual born at time *t*_birth_ [for a derivation of Equation 1 and additional information, see Supplemental Material (doi:10.1289/ehp.1002211) and Ritter et al. 2009]:


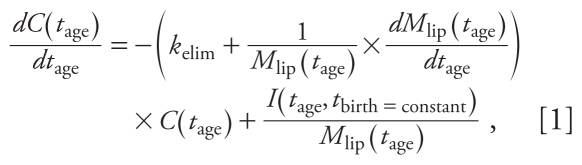


where *t*_age_ (years) is the age of the individual, *C*(*t*_age_) (nanogram per gram lipid) is the lipid-normalized concentration of chemical in the body under the assumption that the chemical is present only in the lipid compartment of the body, *M*_lip_(*t*_age_) (kilograms lipid) is the mass of total body lipid as a function of age, *k*_elim_ (years^−1^) is the first-order rate constant describing intrinsic elimination, and *I*(*t*_age_, *t*_birth_ = constant) (ng × year^−1^ × kg lipid × g lipid^−1^) is the exposure trend of the representative average individual born at *t*_birth_ and is described in terms of age- and calendar-time-dependent daily intake of chemical as


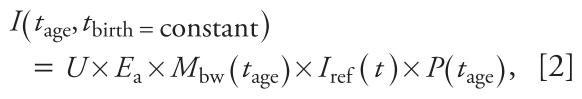


where *U* (days × year^−1^ × kg lipid × g lipid^−1^) is a unit conversion factor selected to describe quantities in commonly reported units; *E*_a_ (dimensionless) describes the net absorption in the gastrointestinal tract and is set at 0.9 (Moser and McLachlan 2001); *M*_bw_(*t*_age_) is the body weight as a function of age in kg; *I*_ref_ (*t*) (ng × kg body weight^−1^ × day^−1^) is the reference daily intake of chemical for an adult and depends on the year of sampling, *t*, which can be expressed as *t* = *t*_birth_ + *t*_age_ (Ritter et al. 2009); and *P*(*t*_age_) (dimensionless) is a proportionality factor adapting *I*_ref_(*t*) to younger ages according to results from total diet studies (Alcock et al. 2000).

Equation 2 describes the daily intake for individuals with *t*_age_ > 6 months, assuming that nursing ends at that age. The daily intake of chemical during nursing is determined from the concentration in the mother [see Supplemental Material (doi:10.1289/ehp.1002211)]. The concentration of chemical in an individual at birth is set equal to the concentration in the mother.

### Estimation procedure

To estimate elimination kinetics, we fit the model to measured data using a least-square optimization by adjusting three fitting parameters: adult reference intakes in the years 1970 and 2000, and *k*_elim_. This is achieved by minimizing the sum of squared residuals weighted (*SSRW*). *SSRW* is related to the coefficient of determination, *R*^2^, of a dataset with *n* empirical data points by


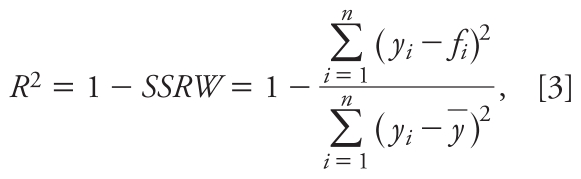


where *y_i_* is a value of the empirical data set, *f_i_* is the associated modeled value, and *y–* is the empirical sample mean. *SSRW* quantifies the differences between modeled and empirical values as the fraction of the sum of squares of residuals (numerator) to the total sum of squares of variability in the dataset (denominator). By minimizing *SSRW*, *R*^2^ is maximized.

For the CSD sets from 1990 and 2003, we define *SSRW*_CSD_1990_ and *SSRW*_CSD_2003_ according to Equation 3. A third *SSRW* value, SSRW_Int_, quantifies the difference between modeled [i.e., *I*_ref_(*t*)] and empirical adult reference daily intake. *I*_ref_(*t*) was defined according to the following assumptions. The intake trend between the years 1970 and 2000 is exponentially interpolated in accordance with monitoring data (Alcock et al. 2002; Bignert et al. 1998). We assume peak intake occurred in 1970. Before 1970, the shape of the modeled adult reference intake trend, *I*_ref_(*t*), is defined relative to intakes in 1970 and 2000 based on information taken from historic emission inventories (Breivik et al. 2002) and exposure reconstruction studies for PCBs. In particular, the intake in the year 1950 is set to the interpolated value of 1990, which is consistent with reconstructed PCB exposures from other studies (Alcock et al. 2000; Karmaus et al. 2004). All three adjustable parameters, the adult reference intakes in 1970 and 2000 and the elimination rate constant, *k*_elim_, are varied independently until the value of an objective function reflecting the differences between modeled and empirical data is minimized. We use two different objective functions: the function *OF*_CSD_Int_ reflects equally weighted contributions of each set of CSD and the empirical intake estimates,


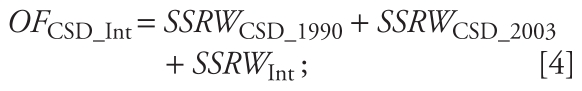


and the function *OF*_CSD_Only_ fits the model by minimizing the difference between modeled and empirical age–concentration CSD only,


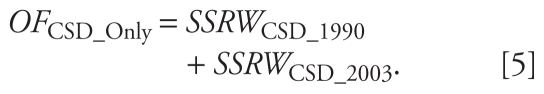


The two different objective functions allow us to test whether it is possible to simultaneously extract information about both exposure and elimination from multiple age–concentration CSD sets alone.

In summary, by minimizing the objective functions *OF*_CSD_Only_ and *OF*_CSD_Int_ in the course of two separated optimization runs, two different sets of results are generated. Each set consists of estimates of the intake in 1970, the intake in 2000, and the rate constant *k*_elim_. Intrinsic elimination half-life estimates are calculated as ln(2)/*k*_elim_.

## Results

Table 2 presents our results along with intrinsic half-life estimates from other studies for the same congeners. Estimates from model fits using *OF*_CSD_Int_ are based on all available empirical information and are therefore judged as best estimates from this study. The shortest intrinsic elimination half-life derived from *OF*_CSD_Int_ is 2.6 years for PCB‐52, and the longest is 15.5 years for PCB‐170.

**Figure 1:**
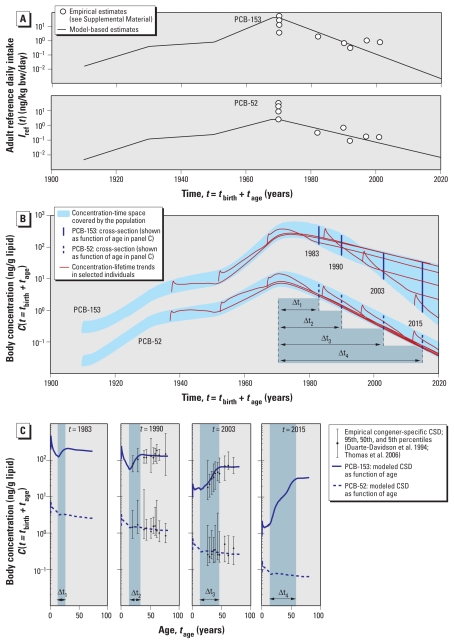
Graphical representation of model fits for PCB‐153 and PCB‐52 from *OF*_CSD_Only_. (*A*) Modeled and empirical adult reference daily intakes. Modeled adult reference intakes were estimated solely by fitting age–concentration CSD as shown in *C*. (*B*) Complete concentration–time space covered by representative individuals of all ages, with examples of concentration–lifetime profiles from six birth cohorts for illustration. Modeled cross sections are indicated as vertical lines. (*C*) Body concentrations of individuals in the four cross sections in (*B*) are plotted as function of age for four different years.

Estimates from *OF*_CSD_Only_ differ by less than a factor of 1.4 from estimates derived from *OF*_CSD_Int_ for all congeners except PCB‐170 and PCB‐180, for which results differ by a factor of 2.1 (Table 2). This good agreement of the results from the two objective functions demonstrates that, in this case, the information contained in multiple sets of age–concentration CSD alone (i.e., using *OF*_CSD_Only_) is sufficient to estimate intrinsic half-lives and to reconstruct historical exposure [Figure 1A; see also Supplemental Material, Figure 2 (doi:10.1289/ehp.1002211)].

Figure 1 graphically presents results of the optimization procedure for PCB‐52 and PCB‐153 using *OF*_CSD_Only_ and illustrates how information about exposure and elimination kinetics is represented in the temporal evolution of the age–concentration CSD. Graphical results for *OF*_CSD_Int_ are almost identical [see Supplemental Material, Figure 2 (doi:10.1289/ehp.1002211)]. Figure 1A shows empirical and modeled estimates of the adult reference daily intake; Figure 1B presents the modeled concentration–time trends for PCB‐52 and PCB‐153 as two shaded areas. Each area represents the range of concentrations for one congener covered by individuals of all ages as a function of calendar time. Within the shaded area, six individual lifetime concentration profiles are highlighted in red as examples. Four cross sections by age are indicated as vertical lines in Figure 1B and plotted as a function of age in Figure 1C.

In the six individual concentration–lifetime trends shown in Figure 1B, the effect of growth dilution is visible as a drop in concentration after weaning at the age of 6 months. This drop is also reflected in Figure 1C in the modeled age–concentration CSD during childhood (i.e., for individuals < 15 years old), which is consistent with empirical data (Toms et al. 2009). Correspondingly, adults are defined as representative individuals > 15 years old because anthropometric data show that body growth becomes much slower around that age (Alcock et al. 2000).

In Figure 1B, the time intervals between the sampling year of each CSD set and the assumed peak year 1970 are labeled Δ*t*_1_ to Δ*t*_4_. These intervals mark the length of the postban periods of declining intake that precede the four CSD age–concentration profiles shown in Figure 1C. Time intervals of the same length are correspondingly labeled in Figure 1C, where they mark the “postban group,” that is, the fraction of the adult population that entered adulthood after 1970. For example, in the age–concentration CSD set from 2003, individuals in the postban group were born between 1955 and 1988, and the representative individual at the right end of the interval Δ*t*_3_ (Figure 1C) is therefore 48 years old. All individuals > 48 years old in 2003 (i.e., born before 1955) were already adults in 1970 and therefore experienced high exposure during the preban period without the mitigating effect of strong growth dilution. As a consequence, for PCB‐153, this preban part of the adult population forms a distinct group showing approximately the same concentration (to the right of the gray-shaded area in Figure 1C). This indicates that PCB‐153 has a relatively long intrinsic elimination half-life that causes a “memory effect” of past exposures. No such memory effect is observed for PCB‐52 because the decline in body concentration for this congener is limited by the rate of decline in exposure rather than the relatively short intrinsic elimination half-life. As a result, average concentrations are similar in all adults for PCB‐52.

The PCB‐153 concentrations increase with age for individuals in the postban group (shaded area in Figure 1C). This is because younger individuals in this group benefit more from the declining exposure trend in the postban period, leading to a smaller memory effect. As the postban period becomes longer (e.g., from Δ*t*_2_ to Δ*t*_3_; Figure 1C), a correspondingly larger part of the adult population shows this increase in concentration with age for PCB‐153. In contrast, for PCB‐52, no increase of concentration with age is evident during the whole adult lifetime; that is, there is no memory effect.

## Discussion

### Intrinsic half-lives

Our results agree well with intrinsic elimination half-lives from two other studies (Table 2). Ogura (2004) used data from the general Japanese population and applied a method similar to ours. Ogura (2004) also accounted for changes in ongoing exposure derived from total diet studies and for age-dependent changes in the size of the body’s lipid compartment by using a PK model. Unfortunately, Ogura (2004) investigated different congeners than in our study, except for PCB‐105 and PCB‐118. For these two congeners, Ogura’s estimated intrinsic elimination half-lives of 5.2 years (PCB‐105) and 6.3 years (PCB‐118) are very similar to our estimates of 5.2 and 9.3 years. Grandjean et al. (2008) used a different approach and investigated intrinsic elimination using LD from a large cohort of children from 4 to 14 years old. They did not employ a PK model but used regression analysis to account for changes in ongoing exposure (i.e., the consumption of whale meat) and for changes in body weight. They achieved this by including the body mass index and the number of monthly whale dinners as covariates in the regression. Grandjean et al. (2008) found no indication that intrinsic elimination half-lives depend on age or are shorter in children than in adults after correcting for the effect of growth. A similar observation has been reported by Yakushiji et al. (1984). Table 2 therefore shows that despite the fact that the half-life estimates are based on different data types (LD and CSD), represent cohorts of different ages, and reflect background levels, consistent estimates of intrinsic elimination half-lives can be obtained if the effects of body weight changes and ongoing exposure are accounted for.

### Contrast between apparent and intrinsic half-lives

Table 3 shows apparent elimination half-lives collected from the literature. Apparent half-lives reflect the overall effect of intrinsic elimination, ongoing exposure, and body weight changes on concentrations as a function of time. Apparent half-lives are subject to a considerably larger variability (Table 3) than are estimates of intrinsic half-lives at background concentration levels (Table 2), which reflect only interindividual variability of intrinsic elimination at similar concentration levels. Apparent elimination half-lives in Table 3 differ by up to a factor of 50 for the same congener. In contrast, the estimates of intrinsic elimination half-lives at background concentration levels in Table 2 differ by less than a factor of 3 and many by less than a factor of 2, although they were derived from different data types (LD and CSD).

The large variability in apparent half-lives reflects cohort-specific differences in all three main factors that influence the observed concentration trend. First, changes in body weight influence the apparent half-life. Growth during childhood leads to growth dilution; that is, much shorter apparent half-lives are observed in infants than in adults (Milbrath et al. 2009). Further changes in body weight during adulthood may also affect the apparent elimination half-life; for example, strong weight loss may even lead to an increase in chemical concentrations with time, which correspond to a negative apparent half-life. In our estimation of intrinsic half-lives, we use a lifetime profile for changes in body weight specific to the U.K. population (Alcock et al. 2002) that takes into account growth dilution during childhood, which is a strong effect in all individuals. For adults, the body weight profile reflects the population average. Second, the rate of intrinsic elimination is faster in cohorts with initial concentrations significantly above background, for example, in incident cohorts measured soon after the exposure incident (Sorg et al. 2009). In such cases, intrinsic elimination reflects the individual status of the patient (e.g., increased elimination via skin, feces, and induced metabolism). In our estimation of intrinsic half-lives, we largely exclude this source of variability by using data from individuals exposed to background concentrations. Third, in cohorts exposed to background concentrations, ongoing exposure can lead to very long apparent elimination half-lives. In our estimation of intrinsic half-lives, we account for this effect by explicitly describing ongoing exposure in the model equation, which allows us to parameterize and quantify intrinsic elimination as a distinct process.

All three effects may contribute to the observation of increasing apparent half-lives in initially highly exposed cohorts that were observed for several decades (Masuda 2001) (Table 3). Because concentrations approach background levels, ongoing exposure becomes relevant also in these incident patients. However, it is not possible to conclude from observed concentration trends whether the increasing apparent elimination half-lives represent a slowdown of intrinsic elimination, for example, due to decreased metabolic activity at lower concentrations (Sorg et al. 2009), or whether it is due to increased confounding from ongoing exposure or body-weight loss.

The strong influence of ongoing exposure and loss of body weight on half-life estimates from incident cohorts that were measured decades after the exposure incident is demonstrated by the observation of very long and even negative apparent half-lives reported for pentachlorodibenzofuran in Yusho patients (Matsumoto et al. 2009). Increasing concentrations (i.e., negative half-lives) can be explained only by additional intake or significant weight reductions under the condition that the substance is not a metabolite synthesized within the body. Very long or infinite apparent elimination half-lives have also been reported for PCBs (Table 3).

The longest intrinsic half-lives from our study are 11.5 years for PCB‐180, 15.5 years for PCB‐170, and 14.4 years for PCB‐153 (Table 2). Other studies (Kreuzer et al. 1997; Shirai and Kissel 1996) have indicated that plausible maximum elimination half-lives of PCBs and dioxins are probably not much larger than 10 years. Our results are further evidence that a maximum intrinsic elimination half-life for persistent chemicals such as PCBs exists and is approximately 10–15 years. This half-life range likely reflects nonmetabolic elimination processes (Kreuzer et al. 1997; Rohde et al. 1999).

### Reducing variability in elimination half-life estimates

Most studies shown in Tables 2 and 3 report “half-lives” or “elimination half-lives” without further specification. However, to make half-life estimates usable, a conceptual and semantic distinction between apparent and intrinsic elimination half-life estimates is needed. This distinction will help to reduce the use of strongly different estimates of elimination half-lives in epidemiologic assessments, where they are needed to parameterize intrinsic elimination in PK models. An example is PCB‐153, for which half-lives selected for use differ by more than a factor of 5, including 5 years (Toft et al. 2008) and 27 years (Verner et al. 2009). Apparent half-lives reflect the overall effect of several factors, including intrinsic elimination, ongoing exposure, and changes in body weight. If ongoing exposure is small relative to concentration levels and body weight is constant, apparent half-lives may reflect intrinsic elimination, but because there is a conceptual difference between intrinsic and apparent half-lives, they will generally also have different numerical values. Importantly, both types of half-life estimates can be derived from both LD and CSD.

### Uncertainty of estimated intrinsic half-lives

To evaluate the intrinsic half-lives obtained with our fitting procedure, we modified the half-lives by a factor of 1.5 and reran the PK model with these modified half-lives. Visual inspection of the results showed that with these modified intrinsic half-lives the calculated body concentrations clearly do not match the data points from the two sets of CSD. This implies that the uncertainty of the estimated intrinsic half-lives is less than a factor of 1.5. This is consistent with the interstudy variability of the intrinsic half-lives in Table 2, which is a factor of 2 or less for eight of the nine congeners and a factor of 3 for PCB‐138. Also the two estimates obtained from our two objective functions, *OF*_CSD_Only_ and *OF*_CSD_Int_, are in good agreement (difference of less than a factor of 1.4 for seven of nine congeners and a factor of 2 for PCB‐170 and PCB‐180). We recommend the estimates based on *OF*_CSD_Int_, because they integrate information from all empirical data sources.

A strength of our approach is that our estimates of intrinsic PCB elimination half-lives have a broad empirical base. In contrast to biomonitoring data types with no or only one temporal dimension (Table 1), multiple sets of empirical age–concentration CSD data can be satisfactorily fitted only if good agreement in both temporal dimensions, within each set of CSD (age) and between different sets of CSD (calender time), is achieved. This is shown in Figure 1C [and further illustrated in Supplemental Material, Figure 1 (doi:10.1289/ehp.1002211)]. The additional dimension of information provided by more than one set of CSD makes it possible to derive intrinsic elimination half-lives directly from the biomonitoring data (objective function *OF*_CSD_Only_).

### Limitations and research perspectives

We did not separate empirical data according to sex because concentration differences between male and female individuals were small in our data sets [see Supplemental Material (doi:10.1289/ehp.1002211)], which is also consistent with results from other cross-sectional studies (Toms et al. 2009). In addition, a separation by sex would have reduced the size of the data set and therefore the precision of the least-square optimization. Correspondingly, we used median anthropometric data for males and females in the model (Alcock et al. 2000). We also did not separate our data according to parity and smoking status because such information is not consistently available for both data sets. Although these factors may influence apparent elimination half-lives (Milbrath et al. 2009), the influence is likely to be small for cohorts at background exposure levels, relative to the strong influence from ongoing exposure and body weight changes. For our data set from 2003 this is substantiated by the lack of significant correlations between parity and concentration in the data set (Thomas et al. 2006). Increasing efforts devoted to biomonitoring provide a promising perspective that time series of even more than two sets of age– concentration CSD that are stratified for factors such as sex, parity, or smoking status will become available for many persistent chemicals. If applied in a consistent conceptual framework that accounts for the influences of ongoing exposure and body weight changes, such stratified CSD may allow researchers to estimate statistical distributions reflecting the interindividual variability of intrinsic elimination half-lives of persistent chemicals.

## Conclusions

Intrinsic elimination half-life estimates are required to translate between exposure and body concentration. A clear discrimination between apparent and intrinsic elimination half-lives helps to explain the high variability in reported elimination half-lives of persistent chemicals in humans. Multiple sets of age–concentration CSD biomonitoring data that represent the general population at background exposure levels, combined with a population PK model, have the potential to provide information about changes in ongoing exposure and intrinsic elimination kinetics of persistent chemicals.

## Figures and Tables

**Table 1:** Types of human biomonitoring data used to estimate elimination kinetics of persistent chemicals.

Biomonitoring data type	Temporal dimensions of concentration changes^a^	Specific experimental conditions	Empirical exposure data required to account for ongoing exposure	Model used	References
1. LD	One temporal dimension (*t*_birth_ = constant = *t* − *t*_age_)	Incident cohorts: occupational, accidental, or experimental cohorts with initial levels above background	None, but only if concentrations strongly exceed background levels^b^	Log-linear regression	Brown et al. 1989; Bühler et al. 1988; Chen et al. 1982; Masuda 2001; Milbrath et al. 2009; Ryan et al. 1993; Wolff et al. 1992; Yakushiji et al. 1984
2. LD	One temporal dimension (*t*_birth_ = constant = *t* − *t*_age_)	Cohorts at concentration reflecting ongoing (background) exposure	Exposure time-trend information for individuals	Log-linear regression	Grandjean et al. 2008
3. Average or median value from a single CSD set	No temporal dimension	Population or cohort biomonitoring studies	One average population- exposure value	Single-individual PK model at steady state	Shirai and Kissel 1996; Geyer et al. 2004; Ogura 2004
4. Multiple averages or median values of CSD^c^ sets (i.e., CSTD)	One temporal dimension (*t*_age_ = constant = *t* − *t*_birth_)	Population or cohort biomonitoring studies	At least one exposure value if postban conditions^d^ apply	Population PK model, time resolved	Ritter et al. 2009
5. Single set of age–concentration CSD	One temporal dimension (*t* = constant = *t*_birth_ + *t*_age_)	Population or cohort biomonitoring studies	Time-trend information of population exposure	Population PK model, time resolved	Van der Molen et al. 2000; Ogura 2004
6. Multiple sets of age–concentration CSD	Two temporal dimensions (*t* = *t*_birth_ + *t*_age_)	Including empirical exposure data (i.e., using *OF*_CSD_Int_)	Time-trend information of population exposure	Population PK model, time resolved	Present study
7. Multiple sets of age–concentration CSD	Two temporal dimensions (*t* = *t*_birth_ + *t*_age_)	Excluding empirical exposure data (i.e., using *OF*_CSD_Only_)	None	Population PK model, time resolved	Present study

a The three time variables (*t*, *t*_birth_, and *t*_age_) are related by *t* = *t*_birth_ + *t*_age_ and therefore reflect only two temporal dimensions of concentration changes.

b At these high concentrations, intrinsic elimination half-lives are not representative for the general population.

c Representing individuals of constant characteristic age (Ritter et al. 2009).

d Individuals included in CSTD have spent their lifetime in a postban phase.

**Table 2:** Estimates of human intrinsic elimination half-lives at background concentration levels (years) for nine PCB congeners.

Data type	PCB‐28	PCB‐52	PCB‐105	PCB‐118	PCB‐138	PCB‐153	PCB‐170	PCB‐180	PCB‐187	Reference
LD (children)			5.4	5.7	3.7	8.4	7.6	9.1	8	Grandjean et al. 2008
Single set of age–concentration CSD (adults)			5.2	6.3						Ogura 2004
Multiple sets of age–concentration CSD (adults; using *OF*_CSD_Only_)	5.6	2.6	4	9.5	8.4	13.8	7.4	5.5	7.8	Present study
Multiple sets of age–concentration CSD (adults; using *OF*_CSD_Int_)	5.5	2.6	5.2	9.3	10.8	14.4	15.5	11.5	10.5	Present study, recommended value

*OF*_CSD_Only_, objective function using information only from empirical cross-sectional data (CSD); *OF*_CSD_Int_, objective function using information from empirical CSD and empirical dietary intake data. Empty cells indicate that no value was reported for the congener.

**Table 3:** Estimates of human apparent elimination half-lives (years) for eight PCB congeners.

Data type/studies	PCB‐28	PCB‐52	PCB‐105	PCB‐118	PCB‐138	PCB‐153	PCB‐170	PCB‐180
LD (adults)								
Brown et al. 1989	1.4		3.9	5.8	6–7	12.4		
Bühler et al. 1988				0.27–0.82	0.88	0.93		0.34
Chen et al. 1982^a^			0.58	0.83	32	47	47	Inf
Chen et al. 1982^a^			0.51	0.77	20	26	71	Inf
Masuda 2001^b^				1.6	4.5	4.2	5.9	6.0
Masuda 2001^b^				17.6	12.8	9.1	18.4	16.7
Ryan et al. 1993^c^				1.1	3.4	3.8	3.9	4.3
Wolff et al. 1992	4.8	5.5	Inf	9.6	16.7	Inf		9.9
Yakushiji et al. 1984	3.0				16.3	27.5		
Extrapolation (infants)								
Milbrath et al. 2009					0.1	0.2		

Inf, infinite.

a Recalculated by Shirai and Kissel (1996).

b Same patients (Yusho) but observations are from different time intervals after the exposure event.

c Median values of three patients. Empty cells indicate that no value was reported for the congener.
